# Efficacy of clomifene citrate combined *Bushen Culuan Decoction* for the treatment of infertility caused by polycystic ovary syndrome: A protocol of systematic review

**DOI:** 10.1097/MD.0000000000020969

**Published:** 2020-07-02

**Authors:** Jing Feng, Xiao-feng Zhang, Jie-ning Ren, Yu-hua Huang, Xin Zheng

**Affiliations:** a Department of Gynecology, 521 Hospital of Norinco Group, Xi’an, Shaanxi, China; b Department of Gynecology, Xi’an Hospital of Traditional Chinese Medicine, Xi’an, China; c Department of Obstetrics, Weinan Central Hospital, Weinan, Shaanxi, China.

**Keywords:** *Bushen Culuan Decoction*, clomifene citrate, efficacy, polycystic ovary syndrome, safety

## Abstract

**Background:**

The aim of this study is to assess the efficacy and safety of clomifene citrate combined *Bushen Culuan Decoction* (CCBCD) in treating infertility caused by polycystic ovary syndrome (PCOS).

**Methods:**

We will carry out this study to identify eligible randomized controlled trials (RCTs) in Cochrane Library, PUBMED, EMBASE, Web of Science, CINAHL, and China National Knowledge Infrastructure from inception to the present. There are no limitations to the language and publication time. We will perform study selection, data extraction, and study quality assessment. If possible, a meta-analysis will be developed to judge the comparative efficacy and safety of CCBCD with other treatments.

**Results:**

The results of this study will summarize current high quality RCTs to provide direct evidence of CCBCD in treating infertility in patients with PCOS.

**Conclusion:**

This study may provide evidence to determine whether CCBCD is effective and safe or not for the treatment of infertility caused by PCOS.

**Study registration:**

INPLASY202050090.

## 1 Introduction

Polycystic ovarian syndrome (PCOS) is one of the most frequency endocrine diseases in females of reproductive age,^[1–4]^ with reported prevalence of 8% to 13% in such population.^[5]^ However, about 70% affected females are still undiagnosed.^[6]^ Its common clinical symptoms manifest as menstrual irregularities, infertility, hirsutism, acne, obesity, and psychological conditions.^[7–10]^

Previous studies found that it is a major cause of anovulatory infertility, and also induces a high risk of complications.^[11–13]^ Although clomifene citrate (CC) is one of the first-line medications that can help induce ovulation in females with PCOS,^[14,15]^ there are still 15% to 40% of such patients who cannot ovulate after CC administration.^[16]^ Thus, it is necessary to explore alternative therapy adjunctive to CC. Fortunately studies suggested that clomifene citrate combined *Bushen Culuan Decoction* (CCBCD) benefit infertility in patients with PCOS.^[17–22]^ However, no systematic review has addressed this topic. Thus, the aim of this study is to appraise the efficacy and safety of CCBCD for the treatment of infertility caused by PCOS.

## 2 Methods and analysis

### 2.1 Study registration

This study was registered on INPLASY202050090. We have reported this study following the guidelines of Preferred Reporting Items for Systematic review and Meta-Analysis (PRISMA) Protocols.^[23]^

### 2.2 Eligibility criteria of study selection

#### 2.2.1 Type of studies

This study includes randomized controlled trials (RCTs) of CCBCD in treating infertility following PCOS. We will exclude all non-RCTs, such as review, non-clinical trials, and uncontrolled trials.

#### 2.2.2 Type of participants

All eligible female adults (aged more than 18 years old) who were diagnosed as infertility caused by PCOS will be included, regardless race, country, and duration of PCOS.

#### 2.2.3 Type of interventions

All eligible patients in the interventional group received CCBCD.

All eligible participants in the control group received any treatment. However, we will exclude patients who also underwent any forms of CC, *Bushen Culuan* Decoction, or CCBCD.

#### 2.2.4 Type of outcomes

The primary outcomes are total ovulation rate and total pregnancy rate. The secondary outcomes are levels of sex hormone (such as luteinizing hormone, follicle stimulating hormone, and androstadiendione), pregnancy loss, ectopic pregnancy, pregnancy and neonatal complications, and adverse events.

### 2.3 Search strategy

We will search electronic databases in Cochrane Library, PUBMED, EMBASE, Web of Science, CINAHL, and China National Knowledge Infrastructure from inception to the present. These databases will be searched for eligible RCTs published without restrictions to language and publication time. The detailed search strategy of Cochrane Library is provided in Table 1. Similar search strategies will be modified for other electronic databases.

**Table 1:**
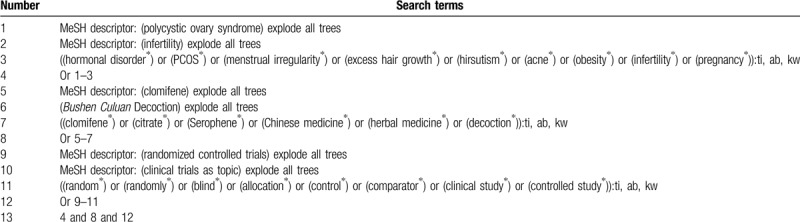
Search strategy of Cochrane Library.

In addition, we will retrieve other sources, such as conference abstracts, ongoing or unpublished studies from clinical trial registry, and reference lists of associated reviews.

### 2.4 Study selection

Two researchers will independently perform study selection by scanning titles and abstracts of all searched citations, and all unconnected studies will be removed. Then, full-text of all potential trials will be read against all inclusion criteria. If there are divergences between two researchers, we will invite a third researcher to discuss and solve the divisions. We will present study selection in a PRISMA flow chart.

### 2.5 Data extraction

Two researchers will independently extract data from eligible trials using pre-piloted, standardized and structured form, and any conflict will be cleared up by a third researcher through discussion. We will extract data of title, first author, country, type of PCOS, number of arms, number of patients, trial setting, trial design, trial methods, details of CCBCD and comparators, outcomes and their measurement time points, results, findings, withdrawals, and adverse events. Any disagreement will be resolved by a third researcher.

We will contact primary trial authors to request any insufficient, unclear or missing data. If we cannot obtain such data, we will perform outcome data analysis using intention-to-treat analysis.

### 2.6 Risk of bias assessment

The study methodological quality of all included RCTs will be assessed using Cochrane risk of bias tool. It has seven domains, and each one is further rated as “high,” “unclear,” or “low” risk of bias. If there are divergences between two researchers, we will invite a third researcher to solve those dissimilarities through discussion.

### 2.7 Statistical analysis

RevMan 5.3 software will be utilized for data analysis. The effect size of continuous data will be estimated using standardized mean difference (MD) and 95% confidence intervals (CIs), and that of dichotomous data will be expressed using risk ratio and 95% CIs. We will apply *I*^2^ statistic to employ statistical heterogeneity. *I*^2^ ≤50% suggests little heterogeneity; and we will pool outcome data using a fixed-effects model; and will carry out a meta-analysis if sufficient data are extracted from included trials. *I*^2^ > 50% exerts remarkable heterogeneity; and we will undertake subgroup analysis and meta-regression to explore possible sources of obvious heterogeneity.

### 2.8 Subgroup analysis

A subgroup analysis will be performed according to the different study information, patient characteristics, study quality, sample size, and outcome indicators.

### 2.9 Sensitivity analysis

A sensitivity analysis will be conducted to test the robustness of study findings by eliminating low quality studies.

### 2.10 Reporting bias

Reporting bias will be identified using Funnel plot and Egger's regression test if over 10 eligible RCTs are included.^[24,25]^

### 2.11 Quality of evidence

Two researchers will appraise the quality of evidence for each outcome utilizing Grading of Recommendations Assessment Development and Evaluation.^[26]^ Any differences will be fulfilled with the help of a third researcher through discussion or consultation.

### 2.12 Ethics and dissemination

This study does not need ethical approval, because it will not collect individual patient data. We will submit this study on a peer-reviewed journal or conference meeting.

## 3 Discussion

PCOS is the leading cause of infertility in women of reproductive age.^[1–4]^ An increasing number of eligible trials reported the CCBCD in treating infertility in patients with PCOS.^[17–22]^ However, no systematic review is performed to appraise the efficacy and safety of CCBCD in treating infertility in patients with PCOS. Thus, the purpose of this study is to summarize the up-to-date clinical evidence of CCBCD in the treatment of infertility caused by PCOS.

To avoid potential bias, this study will examine relevant sources as comprehensive as possible. As to the exploration of potential heterogeneity, subgroup analysis and sensitivity analysis will be conducted. This study will also identify reporting bias to avoid the bias that may affect study findings. The results of this study may provide evidence to help determine whether or not CCBCD is effective and safe in the treatment of infertility caused by PCOS.

## Author contributions

**Conceptualization:** Jing Feng, Xiao-feng Zhang, Yu-hua Huang, Xin Zheng.

**Data curation:** Jing Feng, Jie-ning Ren, Xin Zheng.

**Formal analysis:** Jing Feng, Xiao-feng Zhang, Jie-ning Ren, Xin Zheng.

**Investigation:** Xin Zheng.

**Methodology:** Jing Feng, Xiao-feng Zhang, Jie-ning Ren, Yu-hua Huang.

**Project administration:** Xin Zheng.

**Resources:** Jing Feng, Xiao-feng Zhang, Jie-ning Ren, Yu-hua Huang.

**Software:** Jing Feng, Xiao-feng Zhang, Jie-ning Ren, Yu-hua Huang.

**Supervision:** Xin Zheng.

**Validation:** Jing Feng, Xiao-feng Zhang, Yu-hua Huang, Xin Zheng.

**Visualization:** Jing Feng, Xiao-feng Zhang, Jie-ning Ren, Yu-hua Huang, Xin Zheng.

**Writing – original draft:** Jing Feng, Jie-ning Ren, Yu-hua Huang, Xin Zheng.

**Writing – review & editing:** Jing Feng, Xiao-feng Zhang, Jie-ning Ren, Xin Zheng.
